# On the Origin of Reduced Cytotoxicity of Germanium-Doped Diamond-Like Carbon: Role of Top Surface Composition and Bonding

**DOI:** 10.3390/nano11030567

**Published:** 2021-02-25

**Authors:** Josef Zemek, Petr Jiricek, Jana Houdkova, Martin Ledinsky, Miroslav Jelinek, Tomas Kocourek

**Affiliations:** 1 Institute of Physics of the Czech Academy of Sciences, Na Slovance 2, 182 21 Prague 8, Czech Republic; jiricek@fzu.cz (P.J.); houdkova@fzu.cz (J.H.); ledinsky@fzu.cz (M.L.); jelinek@fzu.cz (M.J.); kocourek@fzu.cz (T.K.); 2 Faculty of Biomedical Engineering, Czech Technical University in Prague, nam. Sitna 3105, 27201 Kladno, Czech Republic

**Keywords:** germanium, diamond-like carbon, photoelectron spectroscopy, low energy ion scattering spectroscopy, Raman spectroscopy, carbon capping film, cytotoxicity

## Abstract

This work attempts to understand the behaviour of Ge-induced cytotoxicity of germanium-doped hydrogen-free diamond-like carbon (DLC) films recently thoroughly studied and published by Jelinek et al. At a low doping level, the films showed no cytotoxicity, while at a higher doping level, the films were found to exhibit medium to high cytotoxicity. We demonstrate, using surface-sensitive methods—two-angle X-ray-induced core-level photoelectron spectroscopy (ARXPS) and Low Energy Ion Scattering (LEIS) spectroscopy, that at a low doping level, the layers are capped by a carbon film which impedes the contact of Ge species with tissue. For higher Ge content in the DLC films, oxidized Ge species are located at the top surface of the layers, provoking cytotoxicity. The present results indicate no threshold for Ge concentration in cell culture substrate to avoid a severe toxic reaction.

## 1. Introduction

Diamond-like carbon (DLC) is a metastable form of amorphous carbon with plentiful interesting mechanical, optical and electronic properties, mainly influenced by the sp^3^/sp^2^ ratio and by the occurrence of hydrogen or other elements. The balance of the components and the incorporation of additional elements can—to certain extent—be tuned to tailor the coatings for a particular application [1,2,3,4]. In biological and medical applications, DLC coatings are often used as barrier layers to prevent corrosion phenomena manifesting themselves by releasing toxic ions from the surfaces of metallic implants into the surrounding tissue [5,6]. The coatings are considered as biocompatible with none or low cytotoxicity. For nanostructured carbon materials, such as graphene, fullerene and nanotubes, the picture is more complicated [7,8]. A weak property of the undoped DLC layers with a high sp^3^ component is a relatively high internal stress resulting in a poor adhesion. This deficiency can be overcome by doping suitable element(s) during a DLC layer deposition. Introducing elements, such as B, N, Ca, P, Ge, Ti, Si leads to improved adhesion mainly due to doping-induced bonding transformation from C sp^3^ hybridization prominent in undoped DLC to C sp^2^ in doped DLC [9]. The next requirement on the doped DLC layers is no or weak cytotoxicity, which should be carefully tested for each dopant element and dopant concentration used.

Germanium is considered to be a nonessential element with low acute toxicity. Ge exerts prophylactic and therapeutic effects in the treatments of cancer and Human immunodeficiency Virus (HIV) infections [10,11,12], immune activation effects for bio-germanium [13], and antioxidant effects [14]. Ge-containing dietary supplements were widely used in the 1970s in Japan and later in other countries to promote health and cure diseases. However, a prolonged intake of Ge-containing products was associated with renal failure with characteristic renal dysfunction and tubular degeneration, anemia, myopathy, neuropathy, tissue Ge accumulation, and even death [15]. Inorganic (GeO_2_) and organic (Ge-132) germanium was found to be toxic to lettuce growth [16]. Cytotoxicity of surface-functionalized silicon and germanium nanoparticles was observed [17]. Germanium nanocrystals doped with boron and phosphorous showed significant toxicity to a Gram-negative bacterium *Shewanella oneidensis*, while the undoped nanocrystals were found to be nontoxic [18]. Antimicrobial properties of a modified multilayer (undoped DLC and Ge-doped DLC coatings with Ge-doped top layer) have been investigated by Robertson et al. [19]. Ge-doped DLC coatings showed a significant anti-biofouling effect on a Gram-negative bacterium *Pseudomonas aeruginosa*. The authors did not attribute this effect to Ge doping alone because *P. aeruginosa* biomass reduction was also observed for the undoped DLC layers [19]. Recently, no cytotoxicity has been followed for a low doping level of Ge-doped DLC layers by Jelinek et al., while for higher doping level the cytotoxicity has been demonstrated, associated with the production of reactive oxygen species. The authors concluded that there is a threshold in Ge cytotoxicity in Ge-doped DLC layers [20].

From the above literature one can infer that Ge can promote health and can be detrimental to health, depending on conditions. Until now, a few reports on Ge-doped DLC layers have appeared. Existing studies mostly provide bulk-like information about Ge-doped carbon layers [19,20]. However, surface properties, e.g., surface composition and surface chemistry, are of critical importance for biocompatibility [5,6]. 

The goal of the present work is to understand the behaviour of Ge-doped DLC layers in relation to the cytotoxicity thoroughly examined in [20]. Especially, we try to reveal the origin of the threshold in Ge-doped DLC layers. Considering that the interaction between a solid surface and tissue is governed by the composition and the chemical bonding of elements located at the top surface and just beneath the surface of the sample, we determined the composition and chemical bonding of atoms located at the surface of the analysed layers by applying two-angle X-ray induced core-level photoelectron spectroscopy (ARXPS) and Low Energy Ion Scattering (LEIS). Raman spectroscopy (RS) was used to validate the bonding transformation from C sp^3^ to C sp^2^ induced by Ge doping deduced from the C 1s spectra.

## 2. Materials and Methods

### 2.1. Deposition

DLC:Ge layers, deposited by the dual pulse laser deposition (PLD) using two KrF excimer lasers, were prepared concurrently with the samples tested for cytotoxicity [20] on substrates from fused silica. More details about the layer deposition can be found elsewhere [20]. In brief, the first laser was focused onto a high purity graphite target, while the second beam was focused on a Ge target. The film thickness reached about 160 nm. An undoped DLC layer was deposited and analysed to reveal the effect of Ge doping in DLC:Ge layers. 

### 2.2. Spectrometers

**Figure 1:**
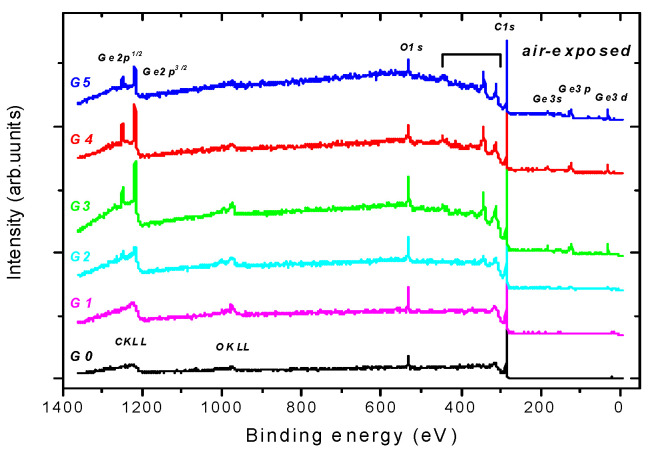
Typical photoelectron survey spectra recorded at normal electron emission angle from the surface of the undoped diamond-like carbon G0 and Ge-doped samples G1-G5. The samples labelling is introduced in Table 1.

Photoelectron spectra were recorded with an AXIS-Supra photoelectron spectrometer (Kratos Analytical Ltd., Manchester, UK), using monochromatized Al Kα radiation (1486.6 eV, 300 W, area analysed—0.7 × 0.3 mm^2^). The samples were analysed ex-situ. Only the peaks of C, Ge, and O were observed in X-ray photoelectron spectroscopy (XPS) survey spectra, see Figure 1. 

The high-energy resolved spectra of C 1s, Ge 3d and O 1s were collected with a pass energy of 10 eV and with a step of 0.05 eV, resulting in an overall energy resolution of 0.45 eV, measured on the width of the Ag 3d_5/2_ line. Binding energy referencing was performed with respect to the C sp^2^ contribution, which peaked at 284.3 eV. Quantification was performed using the integrated peak areas of the C 1s, Ge 3d and O 1s core level spectra after subtracting the standard Shirley‘s electron inelastic background and the atomic sensitivity factors given in ESCApe software, version 1.4 (Kratos Analytical Ltd., Manchester, UK). The C 1s spectra were analysed by peak fitting using the asymmetric pseudo-Voigt peak shape to separate the C sp^2^ bonds [21] and using the symmetric Voigt curves to separate the C sp^3^ contribution and other resolved bonding states in the C 1s and Ge 3d spectral envelopes. LEIS measurements were performed with an AXIS-Supra photoelectron spectrometer (Kratos AnalyticalLtd., Manchester, UK) using ^4^He as incident ions at a scattering angle of 130° and ion energy of 1000 eV. Raman spectra were measured by using Renishaw inVia Reflex microspectrometer, (Renishaw, Wotton-under-Edge, UK) with an excitation wavelength of 442 nm. The total laser power on the sample was decreased to 3 mW so as not to damage the film. The accumulation time was set to 100 s to increase the signal-to-noise ratio. 

### 2.3. Concise Characterization of DLC: Ge Samples

In the recent study of Ge-doped nanocomposite materials dedicated the cytotoxicity [20], the sample properties as surface roughness, morphology, bulk composition and optical transmittance were also examined. Here, we present only brief results important for closer connection the recent and the present studies. Surface roughness ranged from about 3 nm to 120 nm, dependent on Ge concentration, due to small grain droplets located at the surfaces of the Ge-doped samples, as shown by scanning electron microscopy. The Ge dopant concentration averaged within whole layer thickness, measured by a wavelength dispersive X-ray spectroscopy (WDS, JEOL Inc., Peabody, MA, USA), ranged from 0 at. % to 12 at. % (see Table 1). The film transmittance in the UV-VIS and near-infrared regions generally decreased with increasing Ge content. Furthermore, the in vitro adhesion, proliferation, and toxicity of one of very sensitive cell lines (hepatic, Huh7) upon curing on Ge-doped DLC layers using various bioassays were investigated. A threshold for Ge concentration in the layers was identified. Ge concentration higher than 2.5 at. % induced signs of cell death in hepatitis cells. Reactive oxygen species (ROS) production have been identified as a major reason for the cytotoxicity of examined layers. 

## 3. Results and Discussion

### 3.1. Common XPS Surface Characterization

Apparent composition data derived from the C 1s, O 1s, and Ge 3d spectra recorded at the normal photoelectron emission angle from the air-exposed surfaces of the samples, summarized in Table 1, are compared to the bulk-like Ge concentration values averaged within the whole layer derived from a wavelength dispersive X-ray spectroscopy spectra (WDS) [20]. In addition, the content of C sp^2^, C sp^3^ contributions and their ratios is also included. 

**Figure 2:**
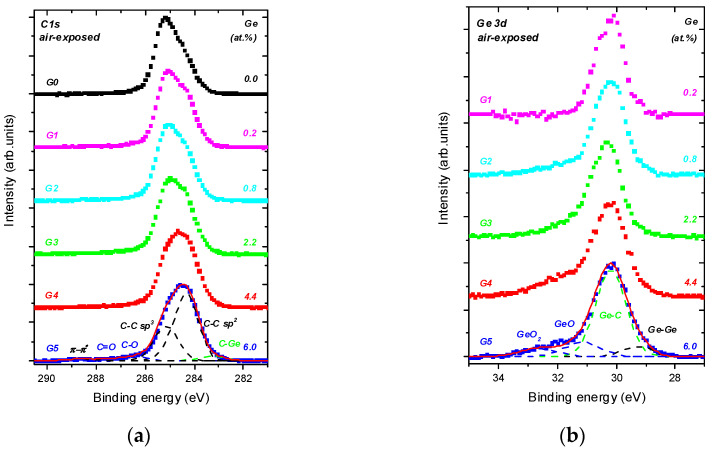
Exemplary high-energy resolved C 1s (**a**) and Ge 3d (**b**) spectra recorded from undoped and Ge-doped DLC layers at the normal emission angle, normalized to unity. Peak-fits of the resolved bonding states are illustrated.

Typical high-energy resolved C 1s and Ge 3d spectra recorded at the normal emission angle from the undoped DLC and the Ge-doped DLC layers are shown in Figure 2. A peak-fit of the C 1s and Ge 3d spectra recorded from the undoped DLC and Ge-doped DLC layers is illustrated. The C 1s envelopes indicate the following bonding states: The spectral intensity at 283.3 eV, ascribed to C−Ge [22,23,24,25,26]; at 284.3 eV, ascribed to C sp^2^; at 285.2 eV, ascribed to C sp^3^ [23,27,28,29,30]; at 286.5 eV, ascribed to C−O; at 287.9 eV, ascribed to C=O [21,31]; at 288.7 eV, ascribed to the π−π* shake up satellite [30]. The Ge 3d envelope indicates the following bonding states: The spectral intensity at 29.3 eV, ascribed to Ge-Ge [25,26]; at 30.2 eV, ascribed to Ge−C [22,25]; at 31.2 eV, ascribed to GeO [32,33]; at 32.7 eV, ascribed to GeO_2_ [32,33]. 

From Table 1 and Figure 2 we deduce that:

(i) In-depth distribution of Ge atoms is non-homogeneous, because the Ge concentrations (WDS) averaged within the layer thickness are larger by a factor of two or even more than the XPS data. As a consequence, a near-surface region of the DLC:Ge samples is partially or fully depleted by Ge.
(ii) The C sp^3^/C sp^2^ ratio is highly influenced by Ge doping, indicating the doping-induced structural evolution of carbon atoms hybridizations from sp^3^ to sp^2^ [9,34]. This is clearly seen in Figure 2a where the dominating C sp^3^ contribution in C 1s envelope for the undoped DLC layer G0 transforms step-by-step to the C sp^2^ one with increasing Ge content in the Ge-doped DLC layers G1-G5.
(iii) Ge−C bonds dominates in Ge 3d envelope over the Ge−Ge and Ge−O bonding states.

### 3.2. Surface Analysis by Using the Methods with Different Information Depths 

As mentioned in the Introduction, the interaction between the solid surface and its surroundings depends on the composition and chemical bonding at the top surface of the solid and by the composition and chemical bonding in a shallow sub-surface region [5,6]. However, it is not an easy task to analyse the top surface of the samples. Widely used surface-sensitive methods, such as XPS and Auger electron spectroscopy (AES), provide information averaged within their information depths (ID). ID represents the thickness of a surface layer from which 95% of the spectral intensity can be recorded. ID can be approximated by ID = 3λ(E)cos α, where λ(E) is the inelastic mean free path (IMFP) of electrons in question, which depends on the electron energy E and the material under analysis, and α is the electron emission angle measured from the surface normal. For example, ID = 6.3 nm is calculated for C 1s photoelectrons travelling in graphite excited by Al Kα radiation recorded at the normal electron emission angle, while for an inclined emission angle of 70° it is ID = 2.2 nm [35,36]. Therefore, ID (and surface sensitivity) changes extensively with the electron emission angle. This effect can be used to obtain qualitative information about the surface and near-surface composition and chemistry [9,27,37]. Similar qualitative information can also be obtained by comparing electron spectra from the same element with the rather different kinetic energy of signal electrons [38]. A more direct and even a more surface-sensitive method is LEIS, known for its extreme surface sensitivity. It provides the composition of 1−2 surface atomic layers [39]. Here we present and compare the XPS spectra recorded at the normal electron emission angle, ID~6 nm; the spectra recorded at an inclined electron emission angle of 70°, ID~2 nm; the LEIS spectra with ID < 1 nm, and finally the RS spectra with ID~100 nm. 

**Figure 3:**
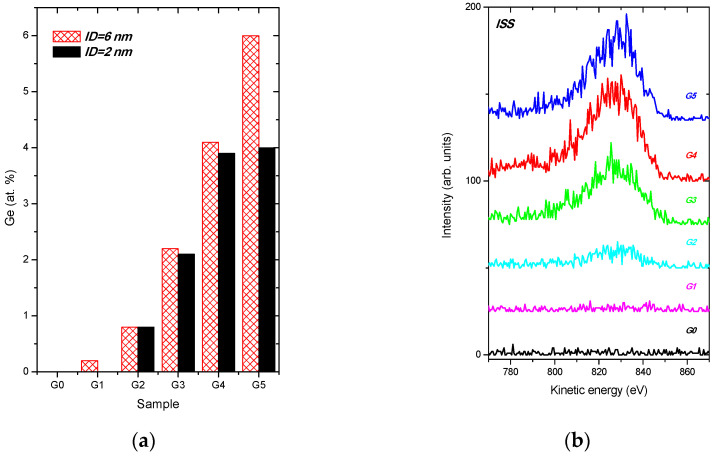
Percentage of Ge species in a surface region of the samples derived from XPS spectra evaluated from ID = 6 nm and 2 nm (**a**). Low energy ion scattering spectra (ID < 1 nm) recorded from undoped DLC G0 and Ge-doped DLC G1−G5 samples, ascribed to 4He ion beam scattering at Ge atoms (**b**).

Germanium content in a near-surface region of the analysed samples, derived from XPS spectra, is shown in Figure 3a for two different ID values. At the more surface sensitive geometry, α = 70°, ID~2 nm, Ge content increased with the sample number and therefore with the bulk-like Ge content summarized in Table 1, and then saturated, while for the α = 0°, ID~6 nm, the Ge percentage is increasing.

Note that no Ge content is found for the G1sample using the more surface-sensitive mode when ID = 2 nm. This means the formation of a carbon capping film covering the surface of the sample. Ge concentration values calculated for both ID values are substantially lesser than the WDS data. This indicates that a near-surface region of the DLC:Ge layers is Ge-depleted, in agreement with the data in Table 1. 

The sensitivity of LEIS for a light element as carbon is relatively poor since the ion scattering cross-sections scale with the atomic number. Moreover, a strong matrix effect occurs in low-energy He^+^ ion scattering from carbon, which causes He ion neutralization [40,41]. As a consequence, the spectral signal of He^+^ ions scattered at carbon atoms is very weak. However, the spectral signal induced by ^4^He ions scattered from Ge atoms can be easily recorded, see Figure 3b. A peak at about 830 eV is ascribed to ion scattering at Ge atoms [39,42]. Indeed, the spectral signal intensity from Ge atoms increased with the Ge content in a surface region of the analysed samples. Their peak areas 312, 941, 1226, and 1293 (in arbitrary units) for the G2−G5 samples, respectively, compare well with the Ge content derived from XPS spectra shown in Figure 3a and in Table 1. We underline that no spectral signal from Ge atoms is detected for the G0 sample (as expected) and for Ge-doped G1 sample. This verifies the formation of the carbon capping film at the surface of the G1 sample, as we already deduced from Figure 3a. The capping carbon film thickness *t* can be estimated in the range of 2 nm ≤ *t* < 6 nm.

**Figure 4:**
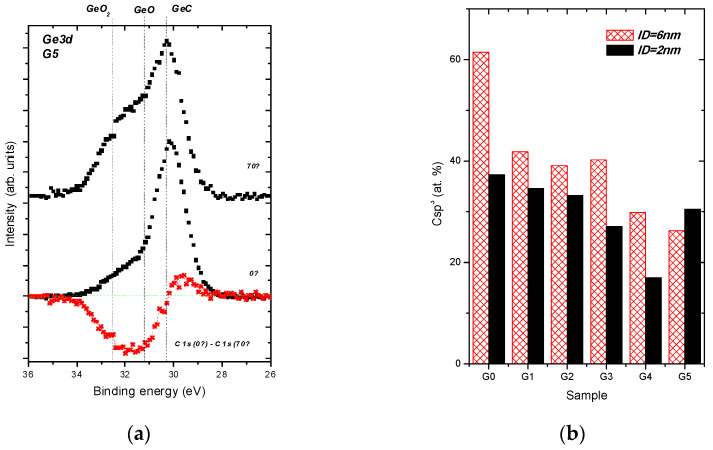
Typical Ge 3d spectra recorded from the DLC:Ge sample G5 at the normal emission angle (ID~6 nm), at the inclined emission angle (ID~2 nm) and their difference (**a**). Content of C sp^3^ contributions for all analysed samples fitted from the C 1s spectral envelopes recorded at the normal and the inclined emission angles and therefore at two different IDs (**b**).

As shown in Figure 2b, chemical bonds of germanium atoms and their in-depth location can be deduced from the Ge 3d photoelectron spectra. The Ge 3d spectra recorded from Ge-doped DLC G5 sample at two different emission angles and therefore at different surface sensitivities and their difference are displayed in Figure 4a. The difference shows marked changes in a region where the spectral signal from Ge−O bonds is expected [32,33]. This indicates that the oxidized Ge species are present at the surface, because the intensity from Ge−O bonds is enhanced in the Ge 3d spectrum recorded within the more surface sensitive mode. Contrary, Ge bonded to C and Ge bonded to Ge are expected to be distributed beneath the surface of the samples [25]. Therefore, the Ge oxide species can easily interact with a tissue to provoke cytotoxicity. Indeed, the Ge oxides, particularly the GeO_2_ can be responsible for the toxic behaviour, as exemplified in previous studies [16,19,43,44]. 

Figure 4b illustrates the next important feature induced by Ge doping—the structural evolution from dominating C sp^3^ component to C sp^2^ component induced by Ge doping examined for both ID values, 6 nm and 2 nm. The C sp^3^/C sp^2^ ratio plays an important role also in the biological activity of carbon materials [8]. The C sp^3^ content decreases and therefore the C sp^2^ content rises with an increasing Ge doping level at the surfaces of the samples, but the G5 sample for ID = 2 nm. The drop is more pronounced for the less surface sensitive geometry, ID = 6 nm, i.e., for a sub-surface region of the samples, because the top surface of the undoped sample is C sp^2^ rich and the C sp^3^ hybridization dominates beneath the surface. The surface enrichment by the C sp^2^ component was observed for the undoped DLC layers prepared by PLD [9] and by other deposition methods [45,46]. Moreover, the surface enrichment by C sp^2^ is consistent with the results of simulated amorphous layer growth [47,48]. Regarding the G5 sample, where the C sp^3^ contribution rose in a shallow surface film of 2 nm, this behaviour can be a sign of a dual structural transformation observed recently for Ca-doped DLC layers. The top surface of the Ca-doped layers is dominantly formed by the C sp^3^ hybridization, while beneath the surface the C sp^2^ contribution is prevailing [9]. 

**Figure 5:**
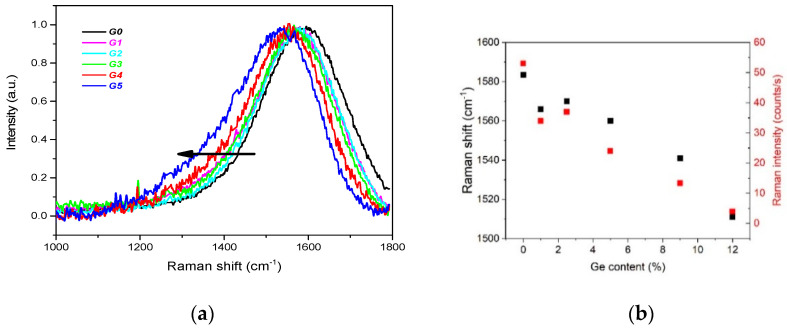
Raman spectra of the analysed samples. The arrow indicates increasing Ge content in DLC:Ge layers (**a**). Changes in the G band position and the Raman signal intensity (**b**) are illustrated.

Two Raman bands are usually characteristic of DLC materials, the D-band and the G-band. For the undoped DLC layer G0 the G band is centred at 1580 cm^−1^, while no D band at ~ 1340 cm^−1^ is detected. This spectral shape is typical of high-quality DLC films [49] and is almost preserved also for the Ge-doped layers (see Figure 5). The G band is shifted to lower energies ~1510 cm^−1^ and, at the same time, getting wider with Ge content. The intensity of the G band significantly decreases with Ge content as well. These are the main signs of an increasing sp^2^ content with Ge doping [22,49], in accord with the above angular-resolved X-ray induced photoelectron spectroscopy (ARXPS) data. 

The C sp^3^ to C sp^2^ structural evolution is frequently observed for metal-doped DLC layers [9,27,34,50]. Foong et al. [34] analysed Cu-doped DLC layers prepared by the laser deposition method. They pointed out that the presence of metal ions during laser deposition increased the heat dissipation on carbon matrix, which enhanced the formation of metallic nano-islands and graphitized the carbon matrix. Due to inversed bremsstrahlung process, the kinetic energy of the metal ions contained in plasma can be higher than in a single carbon system. This idea seems to be reasonable to explain the doping-induced structural evolution from the dominant C sp^3^ to C sp^2^ bonds examined by ARXPS and RS spectroscopy. 

Regarding the cytotoxicity of Ge-doped DLC layers, the present results clearly evidence that their cytotoxicity can be quite high even when the Ge doping is relatively low. The observed missing or low cytotoxicity in [20] can be masked by the capping carbon film formed on the top surface of the samples, when the Ge doping level is low and due to a mixture of Ge oxide and carbon species located at the surface, when the Ge content is higher.

## 4. Conclusions

We analysed hydrogen-free Ge-doped DLC layers using high-energy resolved core-level photoelectron spectroscopy\low-energy ion scattering spectroscopy; and Raman spectroscopy. We proved the formation of the capping carbon film on the top of the low-doped sample that hindered the contact of Ge species with a tissue, thus explaining the absence of cytotoxicity. We next found that the top surfaces of the more doped DLC samples are enriched by carbon species. As a consequence, Ge species are enclosed by carbon atoms and provoked medium or high cytotoxicity, depending on the Ge content. Ge species located at the top surface of the samples were in an oxidized state and can be defined, in agreement with the published data, as the origin of cytotoxicity. Beneath the surface, Ge was dominantly bonded to carbon atoms. The results also confirmed the C sp^3^ to C sp^2^ structural evolution induced by the doping. The present results clearly implicate that the cytotoxicity of the DLC:Ge surfaces can be quite high even when the Ge doping is relatively low. The missing or low cytotoxicity can be masked by the capping carbon film formed on the top surface of the sample, when the Ge doping level is low and due to a mixture of Ge oxide and carbon species located at the surface when the Ge content is higher. Finally, we emphasize the importance of surface-sensitive methods for understanding the interaction between a solid surface and a tissue. 

## Figures and Tables

**Table 1:** Apparent XPS composition data recorded from the undoped DLC and Ge-doped DLC layers. The surface-related contents of the Ge derived from the XPS spectra is compared with the bulk-like values derived from the WDS spectra [20]. The C sp^2^ and C sp^3^ fractions originate from a peak-fitting of the C 1s line, which will be introduced below.

Sample		Ge WDS (at. %)	Ge XPS (at. %)	O XPS (at. %)	C XPS (at. %)	C sp^2^ (at. %)	C sp^3^ (at. %)	C sp^3^/ C sp^2^
G0	Undoped DLC	-	0.0	5.1	94.9	29.9	61.4	2.05
G1	DLC:Ge	1	0.2	3.8	96.0	50.1	41.8	0.83
G2	DLC:Ge	2.5	0.8	4.6	94.6	50.5	39.1	0.78
G3	DLC:Ge	5	2.2	6.8	91.0	42.8	40.2	0.94
G4	DLC:Ge	9	4.1	6.1	89.8	50.3	29.9	0.59
G5	DLC:Ge	12	6.0	7.3	86.7	52.5	26.3	0.50

## Data Availability

Data available on request.
